# Outlook: membrane junctions enable the metabolic trapping of fatty acids by intracellular acyl-CoA synthetases

**DOI:** 10.3389/fphys.2012.00401

**Published:** 2012-10-10

**Authors:** Joachim Füllekrug, Robert Ehehalt, Margarete Poppelreuther

**Affiliations:** Molecular Cell Biology Laboratory, Internal Medicine IV, University of HeidelbergHeidelberg, Germany

**Keywords:** fatty acid uptake, acyl-CoA synthetase, metabolic trapping, membrane junctions, lipid metabolism

## Abstract

The mechanism of fatty acid uptake is of high interest for basic research and clinical interventions. Recently, we showed that mammalian long chain fatty acyl-CoA synthetases (ACS) are not only essential enzymes for lipid metabolism but are also involved in cellular fatty acid uptake. Overexpression, RNAi depletion or hormonal stimulation of ACS enzymes lead to corresponding changes of fatty acid uptake. Remarkably, ACS are not localized to the plasma membrane where fatty acids are entering the cell, but are found instead at the endoplasmic reticulum (ER) or other intracellular organelles like mitochondria and lipid droplets. This is in contrast to current models suggesting that ACS enzymes function in complex with transporters at the cell surface. Drawing on recent insights into non-vesicular lipid transport, we suggest a revised model for the cellular fatty acid uptake of mammalian cells which incorporates trafficking of fatty acids across membrane junctions. Intracellular ACS enzymes are then metabolically trapping fatty acids as acyl-CoA derivatives. These local decreases in fatty acid concentration will unbalance the equilibrium of fatty acids across the plasma membrane, and thus provide a driving force for fatty acid uptake.

Fatty acid uptake and metabolism are exciting areas of research for two main reasons. First, many open questions and conflicting ideas stimulate basic research aimed at elucidating molecular mechanisms and their regulation. Second, lipids are critical for human health, and widespread diseases like diabetes type 2 and atherosclerosis are tightly linked to the dysregulation of lipid metabolism. Here, we take a closer look at a family of essential enzymes which are necessary for the initial stages of fatty acid metabolism. Taking recent experimental evidence into account, we are suggesting a new paradigm for fatty acid uptake based on metabolic trapping across closely spaced intracellular membrane junctions.

## ACYL-CoA synthetases are essential enzymes of lipid metabolism

Long chain fatty acids are the main building blocks of membrane lipids, and as such essential for cellular life as we know it. Furthermore, the oxidation of fatty acids generates two times more energy than other nutrients like sugars or amino acids. Neutral lipids synthesized from fatty acids are efficiently stored in lipid droplets, sustaining life when nutrients in the outside world are diminishing. For all these processes, fatty acids need to be chemically “activated” by the esterification with coenzyme A. The enzymes catalyzing this process are the fatty acyl-CoA synthetases (ACS), and the 26 human enzymes are grouped based on the chain length of their fatty acid substrates (Watkins et al., 2007). The five ACSL and six ACSVL (acyl-CoA synthetase for long/very long chain fatty acids) family proteins are especially physiologically relevant. All of these 11 enzymes will activate common fatty acids like palmitate and oleate; ACSVL proteins are also capable of esterifying the far less abundant fatty acids with 24 and more carbon atoms. ACSVL enzymes are also known as fatty acid transport proteins (FATPs); this functional assignment is mainly based on the correlation between their expression and cellular fatty acid uptake, and the initially reported localization to the cell surface (Schaffer and Lodish, 1994).

## Cellular fatty acid uptake is driven indirectly by intracellular ACYL-CoA synthetases

Our initial studies were focused on the function of FATP4 in fatty acid uptake. Unexpectedly, we found that FATP4 localized to the endoplasmic reticulum (ER) by a variety of independent approaches, including the identification of a novel ER targeting motif (Milger et al., 2006). Nevertheless, overexpression lead to increased cellular fatty acid uptake suggesting an indirect influence of FATP4. A point mutation inactivating the acyl-CoA synthetase activity of FATP4 abolished any effects on fatty acid uptake. This lead us to conclude that FATP4 functions as an ER-localized enzyme, and that this metabolic function is sufficient to increase fatty acid transport. That this could be a general mechanism was supported by our parallel studies on ACSL1 which was localized to mitochondria but also increased fatty acid uptake when overexpressed (Milger et al., 2006). Unfortunately, our results were met with considerable skepticism since other studies had reported a plasma membrane location for ACSL1 and FATP4 (Gargiulo et al., 1999; Stahl et al., 1999). In addition, it was argued that some invisible/undetectable fraction of FATP4 at the plasma membrane could be responsible for all the effects on fatty acid uptake.

Inspired by the differential localization of FATP4 and ACSL1 we then analyzed the subcellular distribution of the other ACS(V)L enzymes (Becker et al., 2007). ACSL3 showed a unique localization to lipid droplets, in addition to the ER. Since ACSL3 had never been observed at the cell surface or suggested to be a transporter protein, this presented us with a fresh opportunity to test our findings again. Depletion of ACSL3 by RNAi diminished fatty acid uptake whereas overexpression had the opposite effect (Poppelreuther et al., 2012). Further work on FATP2 (Krammer et al., 2011) and FATP1 (Zhan et al., 2012) as well as data from other labs (reviewed by Mashek and Coleman, 2006; Digel et al., 2009) are in line with this. Summarizing, we would argue that by now there is sufficient and compelling evidence that *intracellular* acyl-CoA synthetases are indirectly regulating fatty acid uptake.

Cells do not overexpress, knockout or inhibit their acyl-CoA synthetases, which places of course restrictions on the interpretation of the results obtained by these approaches. However, cells and organisms do respond to fasting and refeeding by changing the expression level of ACS(V)L enzymes (e.g., Mashek et al., 2006; Xu et al., 2012). In addition, acute upregulation of the ACS activity of FATP4 by insulin was recently demonstrated (Digel et al., 2011), concomitant with increased fatty acid uptake. We carefully confirmed the localization of FATP4 to the ER after insulin treatment. This was necessary because the related FATP1 enzyme had been suggested to move to the plasma membrane in response to insulin and then start acting to work as a fatty acid transporter (Wu et al., 2006). Since FATP4 remained at the ER, and the enhanced fatty acid uptake was correlated to the wortmannin-sensitive increase of the ACS activity, we finally suggested to use the name ACSVL4 in preference over FATP4.

When we compared the endogenous ACS activity to the fatty acid uptake of C2C12 muscle cells, we were quite surprised to learn that the enzyme activity appears to be in vast excess (50–80×) over what is actually needed to esterify all incoming fatty acids (Digel et al., 2011). Nevertheless, C2C12 cells overexpressing ACSVL4/FATP4 showed a 2-fold increase in fatty acid uptake which corresponded well to the 2-fold higher enzyme activity of these cells (Digel et al., 2011). This suggests that the increased enzyme activity conferred by overexpressed ACSVL4/FATP4 is transmitted to the fatty acid uptake machinery in an attenuated but still proportional manner.

We consider it unfortunate that “transport” and “uptake” are often used synonymously. Here, we use “transport” for the movement of a fatty acid from the extracellular side across the plasma membrane to the cytosol. Uptake comprises both the transport *and* the esterification step which is trapping the fatty acids inside the cell. Therefore, significant *uptake* requires the presence of acyl-CoA synthetases. This was nicely exemplified for adipocytes, where inhibition of the acyl-CoA synthetases by the fungal metabolite triacsin C reduced fatty acid uptake to background levels (Richards et al., 2006). While it is well established that fatty acid binding proteins (FABPs) at the cell surface somehow facilitate fatty acid uptake, the mechanism(s) are not yet clear (Glatz et al., 2010). The term “transporter” is often applied liberally for these proteins even if none of them has been shown to actually transport fatty acids, which tends to confuse researchers outside the field.

## ER-plasma membrane junctions allow efficient lipid transfer across the cytosol

Subcellular membranes differ widely in their lipid composition, and three principal mechanisms account for the sorting and trafficking of the individual lipid molecules: vesicular traffic, diffusion across the cytosol, and exchange mediated by lipid transfer proteins (LTPs). Nonvesicular lipid trafficking is a rising field of research with many divergent ideas, but there is common agreement that lipid transport will only be efficient when donor and acceptor membranes are closely apposed (Levine and Loewen, 2006; Prinz, 2010). The distances observed range from 7 nm to 33 nm, and the structures themselves are termed membrane junctions or membrane contact sites.

The amount of ER-plasma membrane junctions varies among eukaryotic cells but may be extensive (Friedman and Voeltz, 2011). A bridging protein adroitly termed junctophilin has been identified for the highly specialized ER-plasma membrane junction of skeletal muscle cells, but putative structural proteins of membrane junctions in other cell types still await their identification (reviewed by Lev, 2010; Carrasco and Meyer, 2011).

LTPs bind to the two membranes simultaneously or sequentially, and may include a diffusion step across the narrow space as part of their itinerary. Recent overviews show an already impressive list of LTPs grouped into several subfamilies (D'Angelo et al., 2008), but appear to be focused on classical membrane lipids. We would like to suggest here to include the well characterized family of FABPs even if fatty acids are only transient or minor components of cellular membranes. FABPs are small cytosolic proteins and have been reviewed extensively for their lipid transfer capabilities (Storch and Thumser, 2000; Haunerland and Spener, 2004; Furuhashi and Hotamisligil, 2008).

## A new model for the molecular mechanism of cellular fatty acid uptake

Three main models are discussed regarding the mechanism of fatty acid *transport* across the plasma membrane: unassisted diffusion through membranes, protein mediated transport, and protein facilitated diffusion. This has been debated intensely in recent years, but is beyond the scope of our perspective here. Please see (Hamilton et al., 2002; Kampf and Kleinfeld, 2007; Glatz et al., 2010), and references therein.

A current model for the *uptake* process is the vectorial acylation hypothesis which states that transport and activation are functionally coupled to each other. A protein complex located at the plasma membrane has been suggested to promote vectorial acylation (Black and Dirusso, 2003; Richards et al., 2006). One protein would function primarily or even exclusively as a transporter (mammalian FATP1/ACSVL1, or yeast Fat1p), whereas the second protein would be an enzyme trapping the fatty acids by esterification with coenzyme A. We agree that the trapping of fatty acids by acyl-CoA synthetases is a crucial step in the uptake process. However, according to our data the ACSVL/FATP proteins are localized intracellularly to the ER (or to other internal membranes), and therefore cannot transport fatty acids across the plasma membrane. Among the other mammalian ACSL enzymes, ACSL1 has been repeatedly suggested to take over the enzyme function at the plasma membrane localized vectorial acylation complex (Gargiulo et al., 1999; Richards et al., 2006), but we found this protein on mitochondria in many different cell types. In conclusion, our results on the localization of acyl-CoA synthetases in mammalian cells do not support a vectorial acylation complex at the plasma membrane.

**Figure 1:**
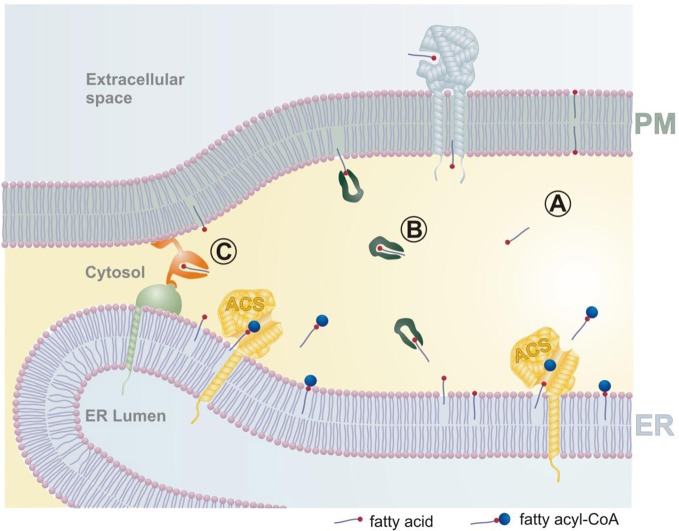
**Membrane junctions enable the metabolic trapping of fatty acids by intracellular acyl-CoA synthetases.** Fatty acids from the extracellular space become integrated into the plasma membrane, which is likely facilitated by plasma membrane associated fatty acid binding proteins (light blue). Transport of fatty acids between the plasma membrane and the endoplasmic reticulum (and other intracellular organelles) may be organized by three different pathways. **(A)** Fatty acids diffuse spontaneously across the narrow membrane junction. **(B)** Cytosolic fatty acid binding proteins (dark green) may act as lipid chaperones to increase transport rates between different membranes. **(C)** Fatty acid transfer proteins (orange) may transport molecules by conformational changes rather than diffusion. Fatty acids delivered to the ER will rapidly equilibrate by lateral diffusion, and finally be esterified by acyl-CoA synthetases (ACS). An increase in ACS activity will lead to a relative depletion of fatty acids from the ER, and this change in the concentration gradient will be transmitted ultimately to the extracellular space, providing the driving force for fatty acid uptake. PM plasma membrane, ER endoplasmic reticulum, ACS acyl-CoA synthetase.

How then do we envisage that fatty acid uptake is organized? As outlined in Figure 1, we suggest that the transport of fatty acids across a cytosolic gap is an essential part of the uptake process. Plasma membrane associated FABPs oriented toward the extracellular side may facilitate the transport of fatty acids by accumulating them close to the lipid bilayer, and CD36 is a candidate for this process (Glatz et al., 2010). Being close to the plasma membrane, the fatty acid will spontaneously integrate into the extracellular leaflet, and flip–flop to the cytosolic side. It is possible that these processes are enhanced by membrane associated proteins. From here on we envisage three different routes for the fatty acid to reach an acyl-CoA synthetase located on a different membrane system. In common, these routes have to be short to be efficient, and this is very likely realized by the formation of membrane junctions between the plasma membrane and the ER. Fatty acids may spontaneously diffuse across the cytosolic space to be integrated in the cytosolic leaflet of the ER (route A). FABPs are protecting the hydrophobic tail of the fatty acids from the aqueous environment of the cytosol and will act as transfer proteins (route B). Finally, dedicated fatty acid transfer proteins anchored to both types of membrane may equilibrate fatty acids by a picking up-delivery mechanism (route C). These possibilities are not mutually exclusive but would differ significantly in the time scales required, with fatty acid transfer proteins offering the fasted transport rates. After reaching the ER, fatty acids will rapidly equilibrate by lateral diffusion. Finally, fatty acids will enter the catalytic site of an acyl-CoA synthetase from the membrane side (Hisanaga et al., 2004; Forneris and Mattevi, 2008). Since the ER is an extensive membrane system in contact with all other subcellular organelles, it may also serve as a “highway” to transport fatty acids efficiently to other membranes (Holthuis and Levine, 2005).

It is important to note that all three routes will try to equilibrate the concentration of fatty acids between the different membrane systems. Consequently, the activation of an acyl-CoA synthetase will transiently deplete fatty acids in the ER membrane. This relative deficiency will be short-lived because of the rapid compensation from the plasma membrane and ultimately from the extracellular environment.

Why such a complicated itinerary? We have to admit that placing a trapping enzyme directly at the plasma membrane appears superior to our model proposed here. However, our experimental data leave no room for this. A benefit of our model is that it offers more options for the regulation of fatty acid uptake. Moreover, ACS family proteins might form complexes with fatty acyl transferases, especially at the ER. This would ensure timely and efficient usage of acyl-CoA molecules, before they are hydrolyzed by thioesterases.

In summary, metabolic trapping of fatty acids at internal membranes, especially the ER will create an intracellular concentration gradient which is the ultimate driving force for cellular fatty acid uptake. It is likely that membrane junctions are required for the efficient transfer of fatty acids between the plasma membrane and the ER. Metabolic trapping by enzymes on intracellular organelles rather than vectorial acylation at the plasma membrane account for the fatty acid uptake of mammalian cells.

### Conflict of interest statement

The authors declare that the research was conducted in the absence of any commercial or financial relationships that could be construed as a potential conflict of interest.
